# CAR-Based Cell Therapy in Head and Neck Cancer: A Comprehensive Review on Clinical Applicability

**DOI:** 10.3390/cancers17132215

**Published:** 2025-07-01

**Authors:** Francesco Perri, Margaret Ottaviano, Miriam Tomaciello, Francesca De Felice

**Affiliations:** 1 Clinical and Experimental Head and Neck Medical Oncology Unit, INT IRCCS Foundation G Pascale, 80131 Naples, Italy; 2 Department of Melanoma, Cancer Immunotherapy and Development Therapeutics, Istituto Nazionale Tumori IRCCS Fondazione Pascale, 80131 Naples, Italy; margaret.ottaviano@istitutotumori.na.it; 3 Department of Radiological Sciences, Oncology and Anatomical Pathology, Sapienza, “Sapienza” University of Rome, 00185 Rome, Italy; miriam.tomaciello@uniroma1.it (M.T.); francesca.defelice@uniroma1.it (F.D.F.)

**Keywords:** head and neck cancer, CAR-T, immunotherapy, immune checkpoint, tumor microenvironment

## Abstract

**Background/Objectives**: Chimeric antigen receptor T-cell (CAR-T) therapy is a novel form of adoptive cellular immunotherapy that involves modifying autologous T cells to recognize and target tumor-associated antigens (TAAs) on malignant cells, independent of major histocompatibility complex (MHC) restriction. Although CAR-T therapy has shown remarkable success in treating hematologic malignancies, its efficacy in solid tumors remains limited, largely due to the lack of tumor-specific antigens and the complexity of the tumor microenvironment. This review aims to explore the rationale for continuing the development of adoptive cellular therapies in head and neck cancer (HNC), offering insights into the diagnostic and therapeutic challenges associated with this heterogeneous group of malignancies. **Methods**: We conducted a comprehensive literature review using the PubMed database to identify relevant studies on the application of CAR-T cell therapy in the management of HNC. **Results**: HNC presented numerous barriers to CAR-T cell infiltration, primarily due to the unique characteristics of its tumor microenvironment (TME). The TME in HNC is notably immunosuppressive, with a lymphocytic infiltrate predominantly composed of regulatory T cells (Tregs) and natural killer (NK) cells. These immune cells typically exhibit low expression of the CD16 receptor, which plays a crucial role in mediating antibody-dependent cellular cytotoxicity (ADCC), thereby limiting the effectiveness of CAR-T cell therapy. **Conclusions**: This comprehensive review suggests a potential clinical applicability of CAR-T therapy in HNC management.

## Simple Summary

Cancer immunotherapy has made significant strides with the development of chimeric antigen receptor (CAR) T cell therapy—an innovative approach in which genetically engineered T cells are designed to recognize and attack tumor-associated antigens (TAAs) on cancer cells, independent of major histocompatibility complex (MHC) recognition. While CAR-T therapy has shown remarkable success in hematologic malignancies, its application to solid tumors, including head and neck cancer (HNC), remains limited. Challenges include a lack of tumor-specific targets and the immunosuppressive tumor microenvironment (TME), characterized by high levels of regulatory T cells (Tregs) and natural killer (NK) cells, which hinder CAR-T cell efficacy. Despite these barriers, emerging research highlights the potential of CAR-T therapy in HNC. This review examines the rationale for adoptive cellular therapies in HNC, with a focus on the diagnostic and therapeutic challenges posed by this heterogeneous group of cancers.

## 1. Introduction

Head and neck cancer (HNC) is the seventh most common malignancy globally, with approximately 947,211 new cases and 482,428 deaths reported annually [1]. This group of cancers encompasses a heterogeneous array of tumors arising primarily in the aero-digestive tract, salivary glands, and thyroid [1]. Major risk factors for HNC include tobacco and alcohol use, while infections with human papillomavirus (HPV) and Epstein–Barr virus (EBV) are strongly associated with increased risk of oropharyngeal and nasopharyngeal cancers, respectively [2]. Current curative treatment strategies for HNC typically involve surgery, radiotherapy, systemic therapy, or multimodal combinations of these approaches. Despite significant therapeutic advancements, prognosis remains suboptimal, especially in patients with recurrent or metastatic disease. The 5-year overall survival rate for HNC remains between 50% and 60% [1,3], highlighting a critical need for novel and more effective treatment strategies.

Immunotherapy has emerged as a significant therapeutic modality, complementing conventional treatments and offering enhanced clinical efficacy against a variety of cancers (melanoma, non-small cell lung cancer, urothelial cancer, Hodgkin lymphoma, renal cell cancer) [4]. Due to proven improvement in overall survival compared with standard treatment, pembrolizumab and nivolumab—two antibodies targeting Programmed Death-1 (PD-1)—are currently approved as first-line regimens in patients with recurrent, unresectable, or metastatic head and neck squamous cell carcinoma (HNSCC) whose tumors express Programmed Death-Ligand 1 (PD-L1) with combined positive score (CPS) ≥1 [5,6].

Chimeric antigen receptor T cell (CAR-T) therapy is an innovative form of adoptive cellular immunotherapy [7]. While it has shown remarkable success in hematologic malignancies, its application to solid tumors remains challenging, largely due to the lack of tumor-specific targets. The identification of suitable antigens in solid tumors, including head and neck cancers (HNCs), continues to be a major obstacle. Given the encouraging results of CAR-T therapy in hematologic cancers, this comprehensive review aims to evaluate its potential role in HNC management. The narrative format is justified given the complexity and interdisciplinary nature of the topic, integrating data from immunology, oncology, molecular biology, and translational research. We explore the complex interactions between the immune system and the HNC tumor microenvironment, highlighting the diagnostic and therapeutic challenges posed by this heterogeneous group of malignancies. Furthermore, we examine the pathophysiological links between CAR-T cells and tumor-associated antigens (TAAs), emphasizing the need for multidisciplinary collaboration to advance treatment strategies and improve patient outcomes in HNC. We review and discuss the current literature of CAR-based cell therapy in HNC to provide a comprehensive summary of recent pre-clinical and clinical trials. Our goal is to better define the potential role of CAR-based cells in treating and controlling HNC in the near future.

## 2. Materials and Methods

A literature review was performed to identify relevant studies on CAR-T cell therapy in HNC management. We conducted a Pubmed search using the following terms equation: “car-t” [All Fields] AND (“head neck” [Journal] OR (“head” [All Fields] AND “neck” [All Fields]) OR “head neck” [All Fields]). The search was limited to papers published in English without any restrictions on publication date. The last search was carried out on 25 March 2025. Trial register (clinicaltrials.gov) and meeting proceedings (European Society of Medical Oncology, American Society of Clinical Oncology and European Society for Radiotherapy and Oncology) were hand searched separately. Reference lists of previously published reviews were explored. Prospective and retrospective studies reporting on CAR-T in HNC were included. An attempt was made to include all relevant studies in humans, in vivo and in vitro.

## 3. Results

In the following sections, (i) we briefly discuss the CARs structure; (ii) we provide an overview of CAR-T applicability in HNC, highlighting various aspects of tumor biology, immune system, and TME; and (iii) we review the preclinical and clinical trials in the HNC field that may influence the future paradigm of care.

### 3.1. CARs Structure

**Figure 1:**
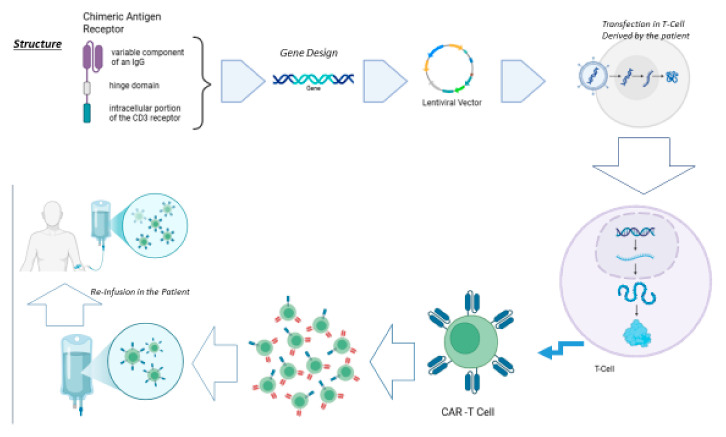
CAR-T cell therapy is based on the creation of specific T cells capable of recognizing a specific antigen towards which the receptor is directed. It starts from the hypothetical structure of the receptor, which, converted into nucleic acid, is transfected into T cells taken from the patient. The latter are re-infused into the patient.

CARs represent a novel immunotherapeutic approach to address autologous cytotoxic T lymphocytes (CTLs) against TAAs, which are expressed on the surface of malignant cells, completely bypassing the entire antigen presentation process, which is dependent on the major histocompatibility complex (MHC) [8]. To generate CAR-T cells, autologous or allogenic T-cells need to be modified to express a CAR. Indeed, the first step is isolating leukocytes from a blood sample from either the patient (autologous) or a healthy donor (allogeneic). The T cells are then separated from the remaining lymphocytes and then transduced with a gene encoding the engineered CAR using a retroviral or lentiviral vector. Alternatively, a transposon can also be used for this process. The first-generation CARs were transmembrane chimeric proteins formed by the fusion between the variable component of an IgG and the transmembrane component of the CD3 receptor of T lymphocytes, obtaining a rapid and highly specific recognition between T cells and TAA as well as an equally rapid signal transduction with relative activation of the T cells (Figure 1). Over time, CARs have undergone substantial evolution aimed to reduce toxicity while increasing their capability to elicit a specific immune response. New-generation CARs are composed by a domain that acts as a “hinge” to allow the previous one to bind to the plasma membrane, a transmembrane domain, and finally an intracellular domain that actually consists of the intracellular portion of the CD3 receptor with an associated costimulatory domain, generally taken from some costimulatory molecules such as CD28, OX40, and CD137 [9,10,11].

**Figure 2:**
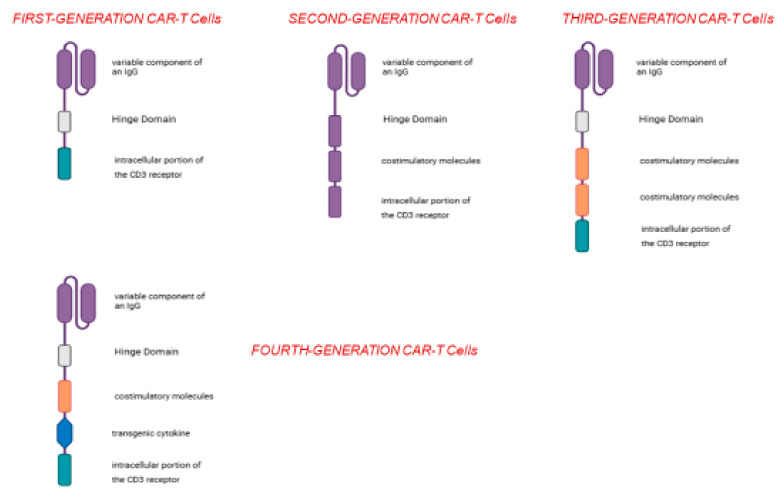
Over time, different types of CAR-T cells have been generated; the first-generation CAR-T cells have a very simple structure and, apart from the variable IGG domain, they possess the intracellular domain of CD3; the second-generation CAR-T cells possess an additional domain, i.e., a co-stimulatory molecule, and their use is accompanied by greater efficacy and a greater probability of eliciting a cancer-specific immune response; the third- and fourth-generation CAR-T cells have more co-stimulatory domains (third generation) and a sequence capable of coding for an immunostimulatory cytokine (fourth generation).

More specifically, based on their structure, CARs have been grouped into five generations [12]. The first-generation CARs, apart from the extracellular portion specific for the TAA, contain a single CD3 intracellular domain. Unfortunately, this type of CAR-T cells was not able to expand and reach a sufficient number to elicit anti-tumor activity [12]. Second-generation CAR-T cells, on the other hand, contain additional cytoplasmic domains encoding co-stimulatory enzymes (such as CD28), which are capable of amplifying the signal and promoting clonal expansion [12]. Third-generation CAR-T cells carry multiple co-stimulatory molecules, including both CD28 and 4-1BB [12]. Fourth-generation CAR-T cells have been engineered to include an additional transgenic cytokine domain—such as IL-7, IL-12, IL-15, IL-18, or IL-23—which significantly boosts the therapeutic efficacy of CAR-T cell treatments [12]. The most recent generation of CAR-T cells is distinguished by the incorporation of IL-2Rβ receptors, which serve as binding sites for STAT3 transcription factors. This enables activation of the STAT/JAK signaling pathway, thereby enhancing both the potency and specificity of CAR-T cells [12] (Figure 2).

### 3.2. Overview of CAR-T Applicability in HNC

The immune checkpoint inhibitors (ICIs) have significantly transformed the treatment landscape of solid tumors, particularly HNSCC [13,14]. These therapies work by blocking immunosuppressive signals exerted by tumor cells—either directly or through modulation of the tumor microenvironment—thereby restoring antitumor immune responses. Despite their promise, approximately 40–50% of patients with advanced HNSCC do not respond to ICIs and eventually experience disease progression. A key mechanism of resistance involves immune evasion through impaired antigen presentation, specifically due to downregulation or loss of MHC expression on tumor cells [15]. In this context, CAR-T cell therapy offers a substantial advantage, as it bypasses MHC restriction, enabling direct recognition and targeting of tumor-associated antigens regardless of antigen presentation status.

Currently, CAR-T cell therapy is approved for use primarily in hematologic malignancies, such as B-cell lymphomas and multiple myeloma, where it has shown encouraging clinical outcomes. However, a growing number of clinical trials are investigating its potential efficacy in solid tumors, including HNSCC [16]. A critical requirement for successful CAR-T cell therapy is the identification of suitable TAAs that are both highly expressed on tumor cells and absent from normal tissues. This is particularly challenging in HNSCC due to its marked heterogeneity and variability in antigen expression. Moreover, the TME in HNSCC is often profoundly immunosuppressive and classified as “immune-excluded” or even “immune-desert,” making CAR-T cell infiltration and efficacy difficult to achieve [17]. One of the major obstacles to CAR-T cell penetration in HNSCC is the presence of a dense peritumoral stroma, which acts as a physical and immunologic barrier to lymphocyte trafficking. Central to this “barrier effect” are cancer-associated fibroblasts (CAFs)—mesenchymal cells that, in response to cytokines such as IL-10 and TGF-β secreted by tumor cells, produce abundant extracellular matrix proteins. These matrix components further impede the infiltration and function of effector T cells within the tumor tissue [18].

### 3.3. CAR-T Cells in Preclinical Trials in HNC

CAR-T cell therapy has demonstrated extraordinary success in hematologic malignancies but continues to face substantial challenges in HNSCC. These include poor infiltration into tumor sites, immune evasion by tumor cells, limited persistence, and the risk of on-target off-tumor toxicity. A multitude of innovative strategies have been developed to overcome these limitations. This section synthesizes current preclinical and translational advances in CAR-T therapy for HNSCC, organized thematically to allow for comparison of approaches.

#### 3.3.1. Enhancing CAR-T Cell Infiltration and Persistence in the Tumor Microenvironment

A critical prerequisite for CAR-T cells to mediate effective cytotoxicity is their ability to reach and infiltrate tumor tissue. To overcome this challenge, several innovative strategies have been proposed [19,20,21]. One approach involves the direct intratumoral injection of CAR-T cells, which has demonstrated enhanced therapeutic efficacy. For example, intratumoral administration of CAR-T cells targeting ErbB+ HNSCC (T1E28z) in a murine model produced superior antitumor effects compared to intravenous delivery of the same cells [22].

Another promising strategy is the combination of CAR-T therapy with photothermal therapy (PTT). Chen et al. developed a protocol wherein CAR-T cells were administered following brief laser irradiation [23]. Elevating the tumor temperature to approximately 44 °C not only induces direct tumor cell damage but also disrupts the tumor vasculature barrier, facilitating CAR-T cell infiltration. Additionally, this thermal modulation promotes the release of pro-inflammatory cytokines such as IL-12 and IFN-γ, further amplifying the antitumor immune response.

Other approaches aim to improve CAR-T activity by engineering them to secrete pro-inflammatory cytokines. Yeku et al. designed CAR-T cells to secrete IL-12, demonstrating that this secretion can overcome TME immunosuppression, promote CAR-T proliferation, and inhibit their apoptosis in ovarian cancer [24].

Engineering CAR-T cells to produce cytokines like IL-23, IL-18, and IL-7, or preconditioning them with IL-15 and IL-7, has shown significant cytotoxic effects against solid tumors [25,26,27,28].

#### 3.3.2. Engineering Immune Function and Resistance to Immune Suppression

Overcoming the suppressive signals of the TME is crucial for CAR-T cell survival and function. Recent studies have engineered CAR-T cells to resist immune checkpoint pathways and improve specificity. Zou et al. engineered CAR-T cells to co-express three immune checkpoint inhibitory receptors, namely PD-1, T-cell Immunoglobulin and Mucin Domain-Containing 3 (TIM-3), and LAG-3, in order to reduce their regulatory effects and prolong CAR-T activity [29]. Similarly, the synNotch receptor system allows for controlled CAR activation upon recognition of a specific antigen combination, enhancing selectivity for tumor cells while sparing healthy tissue [30]. Another strategy involves the use of inhibitory CAR-T (iCAR-T), where the traditional signaling domains are replaced with inhibitory ones, such as PD-1 or CTLA-4, which limit CAR-T proliferation and cytotoxicity against normal tissues [31].

#### 3.3.3. Targeting Tumor-Associated Antigens (TAAs) in HNSCC

Target antigen selection is central to the success of CAR-T therapy. Several TAAs have shown promise in HNSCC.

(i) The Ephrin type-B receptor 4 (EPHB4), a receptor tyrosine kinase, is notably overexpressed in oral squamous cell carcinoma (OSCC), and this overexpression correlates with poor prognosis, making EPHB4 a broadly relevant target in HNSCC and a promising candidate for CAR-T cell therapy [32,33,34,35,36,37,38]. Ito et al. demonstrated that EPHB4-CAR-T cells, especially when administered intratumorally, showed significant tumor regression and T infiltration in both OSCC xenograft and patient-derived xenograft (PDX) models [32].
(ii) Mucin 1 (MUC1) is a transmembrane glycoprotein frequently overexpressed and aberrantly glycosylated in HNSCC, particularly under hypoxic conditions [39]. CAR-T cells specific for MUC1 exhibited strong cytotoxicity, which correlated with MUC1 expression levels [40,41].
(iii) The ErbB family of receptor tyrosine kinases (RTKs)—Epidermal Growth Factor Receptor (EGFR), Human EGFR Related 2 (HER2), Human EGFR Related 3 (HER3) and Human EGFR Related 4 (HER4)—is implicated in HNSCC pathogenesis [42]. EGFR is overexpressed in >90% of HNSCC, and its overexpression is due to a gene amplification. The result of this anomaly is the upregulation of some intracellular pathways related to cell survival, including PI3-K/Akt/mTHOR, JAK/STAT, and Ras/Raf/MEK/ERK-MAPK [43,44,45]. EGFR- CAR-T-cells induced the release of pro-inflammatory cytokines, such as IL-4, IL-10, TNF-α, and IFN-γ, enhancing downstream immune responses [7]. In addition to EGFR gene amplification, overexpression of the EGFR-Her2 heterodimer in HNSCC is also strongly involved in neoplastic progression and the development of distant metastases [46]. HER2 may be used as TAA to engineer specific CARs. Shaw et al. [47] hypothesized that modifying the TME before CAR-T cells injection could increase the efficacy of the treatment. In fact, the authors injected FaDu cells HER2-specific CAR-T cells when combined with a specific oncolytic adenovirus (CAd) encoding the PD-L1-blocking antibody, and IL-12p70 (CAd12_PDL1) produced tumor regression and extended survival in xerograft and orthotopic models [47].
(iv) CD70 is a potent costimulatory molecule and plays a crucial role in immune-system activation, specifically by improving T-cell and B-cell activation, proliferation, and survival. Although they are overexpressed in only 20% of HNSCC, Park et al. showed that CD70-specific CAR-T cells specifically recognized and efficiently eliminated CD70-positive HNSCC cells in vitro [48,49,50].
(v) c-MET is a transmembrane receptor, and its aberrant signal transduction stimulates tumorigenesis with the acquisition of invasive and metastatic phenotypes [51]. c-MET is overexpressed in nasopharyngeal carcinoma (NPC) [52]. Huo et al. demonstrated the efficacy of c-MET targeted CAR-T cells both in vitro and in xenograft models (SCID mice), highlighting its therapeutic relevance in NPC [53].
(vi) CD44v6, a glycoprotein isoform associated with metastasis and tumor progression, is particularly overexpressed in HNSCC, and it can be used as a target for immunotherapy [54,55,56]. Both CAR-T cells and CAR-NK cells targeting CD44v6 showed high efficacy against HNSCC-derived cell lines, with CAR-NK cells offering enhanced safety due to their non-MHC-restricted mechanism of target recognition [57,58].

#### 3.3.4. CAR-NK Cell Therapy: A Safer Alternative

Natural killer cells (NKs) represent a powerful alternative to T cells for CAR-based immunotherapy. Their non-MHC-restricted cytotoxicity reduces the risk of graft-versus-host disease, making them well-suited for allogeneic applications [15,54]. They can elicit antibody-dependent cell cytotoxicity (ADCC). Cetuximab, a monoclonal antibody directed against EGFR, is commonly used in patients with HNSCC both in locally advanced and in recurrent/metastatic settings. Cetuximab is able to elicit the ADCC. Starting from this rationale, Kim et al. tested a novel combination therapy using allogeneic NK cells and cetuximab, both in vitro models and in vivo upon xenograft models of HNSCC [55]. They demonstrated a significant anti-tumor effect of the combination compared to either monotherapy, with high NK cell infiltration and cytotoxic activity in the tumor microenvironment [55]. This simple-design study paved the way to CAR-NK cells therapy in HNSCC.

### 3.4. CAR-T Cells in Phase I Clinical Trials in HNC

Papa et al. carried out a phase I clinical trial enrolling patients with very advanced heavily pre-treated and chemo-refractory HNSCC and administered in them an autologous CD28-based CAR-T cell approach: the T4 immunotherapy [45]. The authors developed a CAR named T1E28z in which the promiscuous ErbB ligand T1E (a chimera derived from Transforming Growth Factor α and EGFR) is fused to a spacer domain and an intracellular domain composed of CD28 and CD3 [45]. Moreover, the authors made possible the coexpression of the receptor T1E28z with the 4αβ chimeric cytokine receptor [7]. In this way, 4αβ may allow interleukin (IL)-4-driven selective expansion of transduced cells during manufacture [45]. This trial was a clear example of fourth-generation CAR-T cells. Both T1E28z- and T4-engineered CAR-T cells demonstrated strong activity in pre-clinical models of several solid tumor types, including HNSCC [7]. This phase I study employed a 3 + 3 dose escalation design and was conducted without lymphodepleting chemotherapy (which is typically required for hematological malignancies). The primary objectives were to identify dose-limiting toxicities (DLTs) associated with T4 immunotherapy and to establish a safe and feasible recommended dose for phase II studies. Secondary objectives included measuring serum cytokine levels following T4 immunotherapy and evaluating both the persistence of CAR-T cells at the injection site and their potential dissemination into the circulation [7]. T4 immunotherapy was administered at a dose ranging from 1 × 10^7^ to 1 × 10^9^ of CAR-T cells, directly by intratumoral injection. Seventeen patients were enrolled, and 15 of them were assessable for response. Overall, stable disease was observed in nine cases, while the remaining presented progressive disease, resulting in a disease control rate of 60% and a median overall survival of 285 days. One out of 15 patients subsequently underwent immunotherapy with pembrolizumab and achieved a partial response lasting 3 years. Regarding the trafficking of CAR-T cells, the authors found that they remained confined to the tumor injection site for approximately 48 h and that there was no spreading of the same into the peripheral circulation. In summary, this study showed that EGFR-restricted CAR-T cells showed fairly good efficacy in cytoreducing the tumor mass following intratumoral injection; however, the absence of spreading into the systemic circulation could correlate with a poor response on any distant metastases [7].

Table 1 shows all the trials employing CAR technique, both pre-clinical and clinical.

## 4. Discussion

This review focused on the potential role of CAR-T therapy in the management of HNC. The therapeutic landscape for HNC is rapidly evolving, propelled by advances in molecular biology, immunotherapy, and drug delivery technologies. While conventional chemotherapy remains fundamental, emerging therapies like CAR-T offer promising new avenues that prioritize both patient survival and quality of life. A critical insight is that identifying TAAs alone is insufficient—especially in HNC, which is characterized by a strongly immunosuppressive TME. Effective CAR-T therapy requires not only targeting appropriate TAAs but also modulating the adaptive immune response to facilitate CAR-T cell activity. Analysis of data from the “whole genome atlas” has helped identify HNSCC-specific TAAs, against which CAR-T cells have been designed and tested in clinical trials.

The use of CAR-T therapy in HNC holds promise, but several challenges remain to be addressed. One of the primary barriers to the successful application of CAR-T in HNC is the complex and immunosuppressive TME. Unlike hematological cancers, where CAR-T cells have shown significant clinical efficacy, solid tumors present challenges such as poor CAR-T cell infiltration and the presence of physical and immunosuppressive barriers. In HNSCC, the tumor microenvironment is often “immune-excluded” or even “immune-desert,” limiting the effectiveness of immune therapies, including CAR-T cells [17]. Furthermore, the highly heterogeneous nature of HNSCC presents an additional challenge in selecting TAAs that are not only tumor-specific but also highly expressed across a significant portion of the tumor. Tumor heterogeneity in antigen expression makes it difficult to find universal targets that would work across all patients, thereby limiting the overall efficacy of CAR-T therapy in HNC. This is particularly critical in HNC, where antigen expression can vary considerably, leading to suboptimal targeting by CAR-T cells [17].

In addition to antigen variability, another significant obstacle is the presence of the peritumoral stroma, which acts as a physical barrier to T cell infiltration. CAFs within the stroma produce large quantities of extracellular matrix proteins in response to cytokines like IL-10 and TGF-β secreted by tumor cells. These proteins hinder the infiltration of T lymphocytes and further contribute to the immunosuppressive environment that limits the success of CAR-T therapy [18].

Recent preclinical and clinical trials have sought to overcome these challenges. Strategies including direct intratumoral injection of CAR-T cells have demonstrated enhanced therapeutic efficacy, as they bypass the barriers of the systemic immune response and directly target the tumor. Combining CAR-T therapy with other modalities—for example PTT—has also shown promise, as it can reduce the physical barriers posed by the tumor vasculature, facilitating better infiltration of CAR-T cells and enhancing their antitumor activity [59]. Moreover, efforts to engineer CAR-T cells to secrete pro-inflammatory cytokines, like IL-12, IL-18, and IL-7, have shown significant promise in inhibiting the immunosuppressive effects of the TME, allowing CAR-T cells to proliferate more effectively and resist apoptosis. Another approach is to design CAR-T cells that can produce cytokines like IL-23 and IL-15 or precondition them with specific cytokines to improve their antitumor responses in the context of solid tumors [25,26,27,28].

The engineering of CAR-T cells to express immune checkpoint inhibitors—PD-1, TIM-3, and LAG-3—is another promising strategy. This allows CAR-T cells to bypass regulatory signals in the TME, increasing their survival and enhancing their ability to infiltrate and attack the tumor [29]. Furthermore, the development of CAR-T cells, which secrete cytokines such as IL-12 to overcome immunosuppression, represents a promising advance in improving CAR-T efficacy in HNC.

The selection of optimal target antigens remains critical for the success of CAR-T therapy in solid tumors. Targets like c-Met, MUC1, CD44, and EGFR are of particular interest in HNC, as they are expressed in a significant proportion of HNSCC tumors. For example, MUC1-targeted CAR-T cells have shown potent cytotoxic activity against HNSCC cells in vitro, while CD44-targeted CAR-Ts have demonstrated efficacy in treating aggressive tumors. Further development of these targeted therapies, as well as the identification of additional tumor-specific antigens, will be crucial to advancing CAR-T treatment for HNSCC [39,53,58].

In addition to enhancing CAR-T cell efficacy, reducing off-target toxicity remains a challenge. Innovations such as the synNotch system, which allows modulation of CAR-T cell affinity for specific antigens, offer the potential for more precise targeting of tumors and reduced damage to healthy tissues [30]. Similarly, iCAR-T systems, which limit CAR-T proliferation and activity through checkpoint signaling, provide a means of controlling the immune response and reducing toxicity [31].

The limitations and feasibility of translating these approaches into clinical practice remain under discussion. From a translational standpoint, several barriers must be addressed. The use of preclinical models that do not fully replicate the complexity of the HNC microenvironment limits the predictive power of early-stage studies. Manufacturing remains labor-intensive and expensive, with variability in CAR-T cell product quality and scalability. Moreover, tumor heterogeneity complicates the identification of robust and universal TAAs suitable for broader patient populations, emphasizing the need for adaptable, perhaps even patient-specific, targeting strategies. On the regulatory front, the landscape is becoming increasingly complex. The clinical trial design for CAR-T therapies in solid tumors—including those for HNSCC—is often constrained by small patient cohorts, inconsistent endpoint definitions, and limited long-term follow-up. The lack of standardized metrics for evaluating immune-related adverse events and therapeutic response creates challenges in regulatory assessment and approval. Regulatory inconsistencies between jurisdictions further complicate global trial harmonization and patient access. The US Food and Drug Administration (FDA) recently issued a warning regarding approved CAR-T cell products following the observation of T cell malignancies in a small number of treated patients. Although the overall incidence remains low—22 cases reported among more than 27,000 patients to date—the recurrence of such events across five of the six approved CAR-T cell therapies prompted the FDA to implement a class-wide boxed warning for these treatments [59]. Emerging strategies that target the insertion of the CAR construct to specific genomic loci may help mitigate the risk of malignancies caused by integration at oncogenic sites. Additionally, comprehensive tumor testing approaches could provide valuable insights into the risk, characteristics, and underlying mechanisms of such therapy-related cancers, further informing safety and patient monitoring efforts.

In conclusion, while CAR-T therapy shows great promise in HNSCC, several challenges must be overcome before it can become a mainstream treatment modality for solid tumors. These challenges include overcoming the immunosuppressive tumor microenvironment, identifying effective tumor-associated antigens, and developing strategies to enhance CAR-T cell infiltration and persistence. Ongoing clinical trials and real-world evidence studies will be crucial in further defining the role of these therapies across the diverse risk groups. As research continues to address existing therapeutic gaps, the future holds promise for the development of more personalized and effective strategies in the management of HNSCC.

## 5. Conclusions

In recent years, our understanding of the role of CARs has improved, and research activity in HNSCC field has increased. However, limited data are still available. Huge scientific effort is needed to advance personalized medicine in the HNSCC patient population. Overall, a key question that needs to be solved soon is which targets are most suitable for which patients. Resolving this could likely lead to better survival outcomes while improving quality of life.

## Figures and Tables

**Table 1:** Pre-clinical and Clinical Trials employing CAR strategy in HNSCC.

	Type	Model	Technique	TAA	Results
Cancer Sci. 2025 Mar 3 [32]	Pre-Clinical	Xenograft	2 generation EPHB4-CAR-T cells	*EPHB4*	Tumor volume reduction
Cancer Med. 2020; 9(2): 640–652. [39]	Pre-Clinical	HNSCC Cell Lines	2 generation MUC1-directed CAR-T cells	*MUC1*	Apoptosis cell lines
J Immunother Cancer. 2023; 11(6): e007162. [46]	Phase I—Clinical Trial	Patients with HNSCC	4 generation T1E28z- and T4-engineered CAR-T cells	*ErbB-ligand (TIE2)*	DCR = 60% mOS = 285 days
Onco Targets Ther. 2018; 11: 7053–7059. [7]	Pre-Clinical	HNSCC Cell Lines	2 generation EGFR-CAR-T-cells	*EGFR*	Apoptosis cell lines
Mol Ther. 2017; 25(11): 2440–2451. [47]	Pre-Clinical	HNSCC Cell Lines	2 generation HER2-CAR-T-cells added to Oncolytic virus ”CAd12_PDL1”	*HER2*	Apoptosis cell lines
Oral Oncol. 2018; 78: 145–150. [50]	Pre-Clinical	HNSCC Cell Lines	2 generation CD70-CAR-T-cells	*CD70*	Apoptosis cell lines
Cytotherapy. 2023; 25(10): 1037–1047. [53]	Pre-Clinical	NPC Cell Lines	2 generation c-MET-CAR-T-cells	*c-MET*	Apoptosis cell lines
Front Immunol. 2023; 14: 1290488. [57]	Pre-Clinical	HNSCC Cell Lines	anti-CD44v6 CAR-NK cells	*CD44v6*	Apoptosis cell lines

## Abbreviations

The following abbreviations are used in this manuscript:

HNC	Head neck cancer
TAAs	Tumor-associated antigens
MHC	Major histocompatibility complex
TME	Tumor microenvironment
T-Reg	Regulatory T cells
NK	Natural killer
ADCC	Antibody-dependent cellular cytotoxicity
EBV	Epstein–Barr virus
HPV	Human papillomavirus
CAR-T	Chimeric antigen receptor T cell
PD-1	Programmed Death-1
PD-L1	Programmed Death-Ligand 1
HNSCC	Head and neck squamous cell carcinoma
CPS	Combined positive score
PRISMA	Preferred Reporting Items for Systematic Reviews and Meta-Analyses
CTLs	Cytotoxic T lymphocytes
ICIs	Immune checkpoint inhibitors
CAF	Cancer-associated fibroblasts
PTT	Photothermal therapy
TIM-3	Immunoglobulin and Mucin Domain-Containing 3
iCAR-T	Inhibitory CAR-T
EPHB4	Ephrin type-B receptor 4
Eph	Erythropoietin-producing hepatocellular
OCSCC	Oral squamous cell carcinoma
PDX	Patient-derived xenograft
IHC	Immunoistochemistry
MUC1	Mucin 1
HIF-1	Hypoxia inducible factor
RTKs	Receptor tyrosine kinases
Cad	Oncolytic adenovirus
HGF	Hepatocyte growth factor
NPC	Nasopharyngeal carcinoma
GvHD	Graft-versus-host disease
DLTs	Dose-limiting toxicities
EGFR	Epidermal growth factor receptor
HER2	Human EGFR Related 2
HER3	Human EGFR Related 2
HER4	Human EGFR Related 4
